# Clinical presentation, evaluation and case management of primary empty sella syndrome: a retrospective analysis of 10-year single-center patient data

**DOI:** 10.1186/s12902-020-00621-5

**Published:** 2020-09-17

**Authors:** Aishah A. Ekhzaimy, Muhammad Mujammami, Shabana Tharkar, Manahel A. Alansary, Daad Al Otaibi

**Affiliations:** 1 Endocrinology and Diabetes Unit, Department of Medicine, College of Medicine and King Saud University Medical City, King Saud University, Riyadh, Saudi Arabia; 2 Prince Sattam Chair for Epidemiology and Public Health Research, Department of Family and Community Medicine, College of Medicine, King Saud University, Riyadh, Saudi Arabia

**Keywords:** Case management, Empty Sella syndrome, Hormone assessment, Saudi Arabia

## Abstract

**Background:**

Primary Empty Sella (PES) syndrome is an increasingly common disorder, mostly diagnosed as an incidental finding during brain imaging scans. We intended to review the clinical management and hormonal profile of patients with PES.

**Methods:**

The study included ten-year retrospective analysis of registry containing PES cases in the period 2007 to 2017, from a single tertiary care center. The keyword ‘primary empty sella’ was used to retrieve patient details from the radiology unit. The clinical and biochemical profile of PES patients was analyzed. Case management of PES patients and their rate of referral to endocrinologists was explored.

**Results:**

The registry had 765 cases with a male: female ratio of 1:3.8 suggesting female predominance by almost four times. Although not significant, the onset of disease was earlier for males [Mean ± standard deviation (SD) (46.7 years ±17.3 vs 48.8 years±14.1), *p* = 0.110]. Almost 79% of the cases were found as an incidental finding during Magnetic Resonance Imaging. Of the total PES cases, only 20% were referred to the endocrinologists and the rest were handled by general physicians. Only 1–2.5% of the cases were evaluated for gonadal, growth and adrenal hormones by the general physicians. The hormonal evaluation by the endocrinologists was also found to be sub-optimal. Headache and visual disturbances were the most common presenting complaints followed by menstrual abnormalities. Endocrine abnormalities like thyroid dysfunction, hyperprolactinemia, hypogonadism and hypocortisolism were highly prevalent among those assessed.

**Conclusion:**

There is a gross under-evaluation of hormonal assessment and minimal case-referral to Endocrinologists. PES is associated with varying degrees of hormonal dysfunction, and hence early assessment and management is needed. Establishing a standard protocol for diagnosis and case management is essential with the involvement of a multidisciplinary team consisting of endocrinologists, neurologists, primary care phys icians and ophthalmologists.

## Background

Empty Sella (ES) is a rare pituitary condition often characterized by the herniated appearance of the pituitary, making it appear flattened with partial or complete filling of the sella turcica space by cerebrospinal fluid. Although the term is referred to as ‘empty’, the pituitary may still function normally. The severity of the syndrome depends on the absence of one or more pituitary hormones due to the extent of involvement of pituitary stalk. The empty sella has been categorized as primary empty sella or secondary empty sella based on the etiology, which is mostly unclear while many hypotheses have been proposed. The congenital absence of diaphragm sellae is the only established etiology for PES, while other causes may include relative changes in intracranial pressure from the cerebrospinal fluid (CSF). And secondary empty sella may result from secondary causes like traumatic factors, side effects of radiotherapy and drugs, infections and necrosis, pituitary tumors or even autoimmune-related [1]. Although the term was first coined by Busch in 1951, Schaeffer, in 1924 first observed the sellae in 125 patients and described the radiological variations from dense to a small peripheral rim of the sellar space [2]. In general, the aetiology of empty sell is not clear and many hypotheses have been studied. The cause of empty sella is either primary (idiopathic) and most likely related to congenital defect in diaphragmatic sella or secondary (acquired) due to pituitary surgery, radiation and pituitary apoplexy. The most common clinical presentation of empty sella syndrome is persistent headache and hormonal deficiencies are rare as clinical presentation for that reason in our paper as in others the diagnosis of empty sella is incidental finding during the work up of neurological symptoms. Since hormonal deficiencies are rare and most likely acquired, the growth and the sexual development less likely to be affected.

Once considered a rare disorder, diagnosis of ES has been increasing since it is primarily regarded as an incidental finding on brain imaging scans like Magnetic Resonance Imaging (MRI) predominantly, Computed Tomography (CT), or rarely plain X-ray. However, very often the patient may present with various symptoms which are attributed to PES in the absence of any other likely causation. The clinical significance related to the incidental finding is ambiguous and some studies elucidate it to more than a mere incidental finding [3]. Several isolated case reports and case series have been published over the last three decades [4] and statistics collectively from single-centerr or multicenter retrospective studies have reported prevalence as low as 2.3% [1] to as high as 38% [5] in autopsy studies with large predominance in the female gender.

Studies from the Saudi Arabian region are scanty except a report from the western region documenting a brief description of 537 patients with PES without exploring further details on clinical significance, the symptoms and hormonal assessments Moreover, no specific guidelines or recommendations exist in the diagnosis and management of PES, resulting in the majority of the cases being managed without a referral to an endocrinologist. However, experts in the field have advised endocrine assessment of even the asymptomatic cases and a multi-disciplinary approach in the management of PES [6]. The objective of the present study is to report a 10-year retrospective review of primary empty sella from a single-center registry, aiming to determine the epidemiology, clinical presentation and management of PES, and to identify the differences in investigations and diagnosis of PES between endocrinologists and non endocrinologists in a tertiary care setting.”

## Methods

Ten-year retrospective data of PES cases was extracted and analysed from the radiology and imaging unit of the tertiary care referral hospital.

Data extraction: The study population comprised of patients who underwent MRI of brain at the radiology unit from 1st July 2007 to 30th June 2017. The keyword ‘Primary Empty Sella’ was used to extract the data. A total of 26,218 patients had undergone MRI in this period and 765 patients were diagnosed to have PES. The most common indications for MRI of the brain included headache, traumatic brain injury, neurological symptoms like stroke and Transient Ischemic Attack. Diagnosis of PES was made if there was i) enlargement of the sella turcica, ii) filling of the sella turcica with CSF and iii) compression and flattening of the pituitary gland against the sella floor. The medical identification number of the PES patients was retrieved to include further details of clinical presentation, hormone profiles and whether the diagnosis was an incidental finding. Upon an incidental finding, the cases were subjected to confirmation by biochemical investigations.

To define hormonal deficiency for each axis, the case would be termed ‘affected’ if: gonadal hormonal deficiency was based on low testosterone in male or estradiol in female with low or inappropriately normal LH and FSH level, the thyrotrophic axis deficiency was defined based on low free T4 and low or low normal TSH level, for corticotroph axis to be considered affected if morning plasma cortisol is less than 100 nmol/l and confirmed by failure to respond to ACTH stimulation by short synacthin test, somatotroph axis was considered affected if IGF-1 level is low for the age-adjusted range in the presence of 3 or more affected axes. The description of each hormonal deficiency is tabulated accordingly;

**Table:** 

**Hormones**	**Normal range**		Remarks
ACTH	AM 1.6–13.9 pmol/L	PM 1.6–11.1 pmol/L	
Random Cortisol	193–690 nmol/L		
TSH	0.25–5 mIU/L		Central hypothyroidism is confirmed if TSH level is low or normal and Free T4 is low.
FT4	11.5–22.7 pmol/L		
LH	Males (20–70 y.o.) 1.5–9.3 IU/L	• Females, normal menstruation, follicular phase 1.9–12.5 IU/L • Females, normal menstruation, mid-cycle peak 8.7–76.3 IU/L • Females, normal menstruation, luteal phase 0.5–16.9 IU/L	In Females, central hypogonadism is confirmed if serum estradiol level is low and LH and FSH is either inappropriately normal or low in both premenopausal and postmenopausal women. In males, central hypogonadism is confirmed if total testosterone level is low and LH and FSH is either inappropriately normal or low as an abnormal response of the pituitary gland
FSH	Males 0.8–9 pmol/L	• Females, normal menstruation, follicular phase (− 12 to − 4) 3–12 pmol/L • Females, normal menstruation, mid-cycle peak (− 3 to + 2) 6–25 pmol/L • Females, normal menstruation, luteal phase (+ 4 to + 12) 2–12 pmol/L • Post-menopausal females 12–30 pmol/L	
Estradiol	Males 43.6–146 pmol/L	• Females, normal menstruation, follicular phase (− 12 to − 4) 71.6–529.2 pmol/L • Females, normal menstruation, mid-cycle peak (− 3 to + 2) 234.5–1309.1 pmol/L • Females, normal menstruation, luteal phase (+ 4 to + 12) 204.8–786.1 pmol/L	
Total Testosterone	Male • 20–49 y.o 8.64–29 nmol/L • > = 50 y.o 6.68–25.7 nmol/L	Female • 20–49 y.o 0.29–1.67 pmol/L • > = 50 y.o 0.101–1.42 pmol/L	
IGF-1	54–204 ng/mL		
Prolactin	Males 45–375 mIU/L	• Non-pregnant females 59–619 mIU/L • Pregnant females 206–4420 mIU/L • Post-menopausal females 38–430 mIU/L	

Hormonal assays: The hormonal profile included assessment of growth hormone (GH), Insulin-like Growth Factor (IGF-I), Thyroid Stimulating Hormone (TSH), Free Thyroxine (FT4), Prolactin, Luteinizing Hormone (LH), Follicle Stimulating Hormone (FSH), Total testosterone in males and Estradiol level in females, Adrenocorticotropic Hormone (ACTH) and Cortisol. Anti-Diuretic Hormone (ADH) deficiency was assessed based on history and biochemical profile (Serum sodium, urine and serum osmolality, 24 h urine input and output). LH, FSH and testosterone were assessed using ADVIA Centaur LH assay kit # 01756298 Siemens, FSH assay kit# 04912924 Siemens and ADVIA Centaur Testosterone II assay kit# 10696862 Siemens respectively. Prolactin and cortisol by ADVIA Centaur Prolactin assay kit# 09505871 Siemens and ADVIA Centaur Cortisol assay kit# 10994926 Siemens. The assay kits used for assessment of FT4 and TSH were ADVIA Centaur FT4 assay kit# 06490106 Siemens and ADVIA Centaur TSH3-Ultra assay kit # 06491080 Siemens. Elecsys ACTH, Roche Diagnostics, Indianapolis, IN V 11.0, 05/2018 kit# 03255751 190 was used to assess ACTH by electrochemiluminescence immunoassay “ECLIA” method. Similarly, IGF-1 utilized Elecsys IGF-1, Roche Diagnostics, Indianapolis, IN V 4/2018 KIT#**0**7475896 190 assay kit. The hormone assays were evaluated by a two-site sandwich immunoassay using direct chemiluminometric technology.

The results of the assays are entered in the centralized electronic health record system of the hospital that can be accessed by all those physicians and healthworkers who are authenticated to its use. Patients who had deficiencies in hormonal profile were referred to as ‘affected. Those who had no evidence of hormonal deficiency despite proven to have PES radiologically were termed as ‘not affected’. Only some cases were referred to the endocrinologists for complete hormonal evaluation.

Ethics approval: The study approval was obtained by the Institution’s Review Board (IRB), College of Medicine, with a reference number of 19/067/IRB-CM. No disclosure of patient identity was made and the data collected was meant solely for the purpose of research.

Statistical analysis: Data was analyzed using the software - Statistical Package for Social Sciences version 22.0. The hormonal profile was reported as mean and standard deviation. Frequencies and percentages were used to report the prevalence of anomalies and clinical symptoms. The differences were tested by chi-square test of proportions or student t-test whichever appropriate and *p*-value below 0.05 was considered significant.

## Results

The registry had enrolled 765 patients with PES in the 10-year period since 2007. Higher female preponderance was noted with 607 females and 158 males. The clinical presentation of PES patients is shown in Table 1. The mean age at diagnosis of the study patients was 48.4 ± 14.8 years and the men showed a lower mean age than women. Occurrence of PES was more common in both male and female patients older than 50 years. Younger males aged less than 29 years showed a higher prevalence of PES than females of the same age. Although it did not reach statistical significance, women presented with symptoms more often than men. While 79% of the cases were diagnosed as incidental findings during MRI scans, only 20% were referred to and seen by the endocrinologists. Further analysis showed that almost 42% of the incidental cases were symptomatic. The most common symptoms reported were headache, visual disturbance including diplopia, nystagmus, fatigue, galactorrhea, infertility and menstrual-related problems. Men had significantly higher gonadal disorders like infertility and diminished libido. Other reported non-specific symptoms include tinnitus, nausea, lack of growth, dizziness or vertigo, seizures, weakness/numbness of head and neck or extremities or body sides, acute behavioral changes, ataxia and dysarthria, delusion, memory loss and myalgia.

**Table 1:** Gender differences at first clinical presentation of PES patients

	Total (765)	Male (158)	Female (607)	*p* value
Mean age (years) (Mean ± SD)	48.4 ± 14.8	46.7 ± 17.3	48.8 ± 14.1	0.110
Gender prevalence	–	158 (20.5)	607 (79.4)	**0.000**
Age-wise distribution:				
≤ 29 years	87 (11.4)	30 (18.9)	57 (9.4)	**0.022**
30-49 years	303 (39.6)	56 (35.2)	247 (40.7)	
≥ 50 years	375 (49.0)	72 (45.3)	303 (49.)	
Asymptomatic	449 (58.6)	103 (64.8)	346 (57)	0.124
Incidental finding	605 (79)	121 (76.1)	484 (79.7)	0.185
Seen by endocrinologist	155 (20.2)	42 (26.4)	113 (18.6)	**0.019**
Common symptoms:				
Headache	227 (29.6)	29 (18.3)	198 (32.6)	**0.001**
Vision disturbance	109 (14.2)	18 (11.3)	91 (15)	0.297
Fatigue	33 (4.3)	7 (4.4)	26 (4.3)	0.596
Galactorrhea	6 (0.8)	–	6 (1.0)	
Amenorrhea	5 (0.7)	–	5 (0.8)	
Irregular cycle	11 (1.4)	–	11 (1.8)	
Infertility	10 (1.3)	6 (3.8)	4 (0.7)	**0.003**
Loss of libido	7 (1.0)	5 (3.1)	2 (0.3)	**0.001**

Data is presented in number (%)

The denominator for percentage calculation is based on gender total (Male158 and female: 607)

**Fig. 1:**
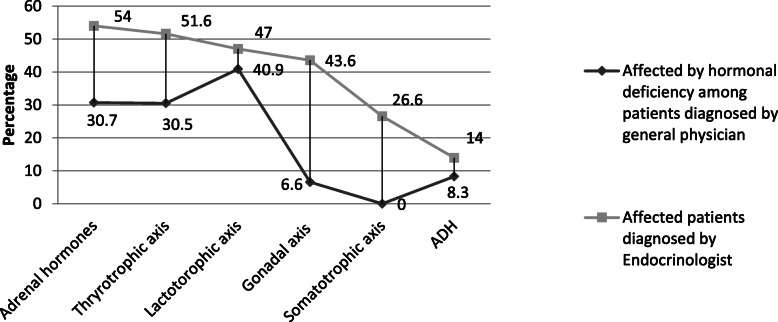
Comparison of those affected by hormonal deficiency between general physician and endocrinologists

Table 2 shows the details of evaluation and clinical management of the patients. The general physicians handled 80% of the cases, and of them, more than 90% were not assessed for gonadal, growth and adrenal hormones. Furthermore, the details of those affected by the hormonal deficiencies are also displayed. Although the endocrinologists had only 20% of the total PES cases, hormonal assessment was still markedly under-evaluated. In addition, the affected among those assessed are summarized in decreasing order of prevalence in Fig. 1.

**Table 2:** Differences in assessment of hormonal profile evaluation between General Physician (GP) and Endocrinologist

	Seen by General Physician	Seen by Endocrinologist
	*N* = 610	*N* = 155
	n(%)	n(%)
**Gonadal axis**		
Not assessed	595 (97.5)	74 (47.7)
Assessed:	15 (2.5)	81 (52.9)
Affected	1 (6.6)	37 (43.5)
**Somatotrophic axis**		
Not assessed	609 (99.8)	140 (90.3)
Assessed:	1 (0.2)	15 (9.7)
Affected	–	4 (26.6)
**Lactotrophic axis**		
Not assessed	588 (96.4)	72 (46.5)
Assessed:	22 (3.6)	83 (53.6)
Affected	9 (40.9)	39 (47.0)
**Adrenal hormones**		
Not assessed	597 (97.9)	92 (59.4)
Assessed:	13 (2.2)	63 (40.6)
Affected	4 (30.7)	34 (54.0)
**ADH**		
Not assessed	248 (40.7)	25 (16.1)
Assessed:	362 (59.4)	129 (83.2)
Affected	30 (8.3)	18 (14.0)
**Thyrotrophic axis**		
Not assessed	423 (69.3)	33 (21.3)
Assessed:	187 (30.6)	122 (79.0)
Affected	57 (30.5)	63 (51.6)

The denominator for percentage is the total sample seen

Affected is calculated within those assessed

*ADH* Anti diuretic hormone

**Fig. 2:**
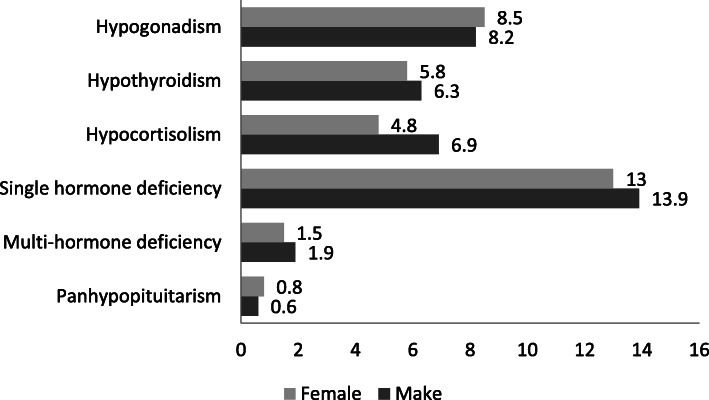
Prevalence of pituitary hormones dysfunction. * Data is reported in percentage

Table 3 provides the general biochemical profile of the PES patients under hormone replacement therapy. Table 4 illustrates the distribution of pituitary hormone levels in PES patients, in addition to the assessment statistics of individual hormones. The categorization of values as low, normal and high is based on reference range, where low and high values are referred to as abnormal. Pituitary dysfunction is referred to two to three hormonal deficiencies (multi-hormonal deficiency) or three or more hormonal deficiencies (panhypopituitarism) as presented graphically in Fig. 2. Males showed higher levels of pituitary dysfunction than females. Hypogonadism was the most frequent disorder observed, followed by hypothyroidism and hypocortisolism.

**Table 3:** General biochemical profile of the PES patients

Hormones	Mean ± SD of those assessed	Normal range*
**Estradiol** (Female only)(pg/mL)		
Pre-menopause	242 ± 205	15–350
Post-menopause	33 ± 20	0–45
**Luteinizing Hormone**IU/L		
Male	3.5 ± 2.0	2.9–6.8
Female		
Pre-menopause	6.7 ± 5.1	1.1–9.2
Post-menopause	10.3 ± 13.4	15–53
**Follicle Stimulating Hormone**IU/L		
Male	3.7 ± 3.9	1.3–11.8
Female		
Pre-menopause	6.4 ± 5.4	2.8–10.2
Post-menopause	29 ± 25.3	13.9–102.1
**Testosterone** (male only)nmol/L	15 ± 13	10–27
**Prolactin** mIU/L		
Male	353.4 ± 335	53–360
Female	429.7 ± 412.8	40–530
**IGF-1 ng/ml**		
Male	141 ± 106	53–461
Female	189 ± 176	43–436
**ACTH** pmol/L	5.6 ± 4.9	1.6–13.9
**Cortisol** nmol/L	327.2 ± 240.9	171–536
**Short Synacthen Test** nmol/L	399.3 ± 269.6	> 500
**TSH** mIU/L	2.6 ± 1.8	0.35–4
**FT4** pmol/L	14.4 ± 4.4	10.2–22.7

^*^Normal ranges taken from Institution’s laboratory reference ranges as per standardized guidelines

*IGF-1* Insulin-like growth factor-1, *ACTH* Adrenocorticotropic hormone, *TSH* Thyroid stimulating hormone, *FT4* Free thyroxine

**Table 4:** The clinical profile of pituitary hormone levels of PES patients

	Male (158)	Female (607)	
	n(%)	n(%)	
**Follicle Stimulating Hormone**	Assessed 22 (14)	Pre-menopause (322) Assessed 35 (10.9)	Post-menopause (285) Assessed 27 (9.4)
Low	7 (31.8)	9 (26.5)	17 (63)
Normal	14 (63.6)	18 (52.9)	10 (37)
High	1 (4.5)	7 (20.6)	–
**Luteinizing Hormone**	Assessed 18 (11.3)	Pre-menopause(322) Assessed 35 (10.9)	Post-menopause(285) Assessed 24 (8.4)
Low	7 (38.9)	4 (11.4)	18 (75)
Normal	10 (55.6)	23 (65.7)	6 (25)
High	1 (5.6)	8 (22.9)	
**Testosterone**	Assessed 30 (18.9)	–	
Low	9 (30)		
Normal	18 (60)		
High	3 (10)		
**Estradiol**	–	Pre-menopause(322) Assessed 14 (4.3)	Post-menopause(285) Assessed 3 (1.1))
Low		–	
Normal		10 (71.4)	2 (66.7)
High		4 (28.6)	1 (33.3)
**Prolactin**	Assessed 28 (17.6)	Assessed 84 (13.8)	
Low	3 (10.7)	8 (9.5)	
Normal	15 (53.6)	39 (46.4)	
High	10 (35.7)	37 (44)	
**Thyroid Stimulating Hormone**	Assessed 70 (44)	Assessed 233 (38.4)	
Low	3 (4.3)	13 (5.6)	
Normal	53 (75.7)	160 (68.7)	
High	14 (20)	60 (25.8)	
**FT4**	Assessed 64 (40.5)	Assessed 224 (36.9)	
Low	8 (12.5)	24 (10.7)	
Normal	54 (84.4)	192 (85.7)	
High	2 (3.1)	8 (3.6)	
**Cortisol**	Assessed 22 (13.8)	Assessed 46 (7.6)	
Low	4 (18.2)	14 (30.4)	
Normal	16 (72.7)	24 (52.2)	
High	2 (9.1)	8 (17.4)	
**Short Synacthen Test**	Assessed 15 (9.4)	Assessed 30 (4.9)	
< 500 nmol/L	8 (53.3)	20 (66.7)	
High	7 (46.7)	10 (33.3)	
**ACTH**	Assessed 16 (10.1)	Assessed 27 (4.4)	
Low	–	8 (29.6)	
Normal	14 (87.5)	17 (63)	
High	2 (12.5)	2 (7.4)	
**IGF-1**	Assessed 6 (3.8)	Assessed 5 (0.8)	
Low	1 (16.7)	–	
Normal	5 (83.3)	4 (80)	
High	–	1 (20)	

All the statistics are in frequency (percentage). The percentage is calculated by taking denominator as within those assessed

Menopausal age considered 51 years

For reference range please refer to Table 3

Low, normal, and high are categorized according to the lab reference range. Therefore, low and high values indicate abnormal. To be noted that ‘normal’ includes those on hormone replacement therapy

Other related disorders: Behcet syndrome was found in four patients and two patients were diagnosed with Woodhouse-Sakati syndrome. There were two cases with high levels of prolactin indicating a diagnosis of prolactinoma.

## Discussion

The present study analyzed the clinical presentation and management of the PES patients. The clinical picture of patients with PES is quite intricate due to the involvement of the extent of hormones from none to all. Since the causation mechanism is not established, it is not clear if the symptoms are due to PES or coincidental. There is extreme variation in the prevalence of endocrine abnormalities reported with PES ranging from normal pituitary function to certain grades of pituitary dysfunction depending on the pituitary stalk compression by CSF or intracranial hypertension. Except for few comprehensive reviews reporting the clinical diagnosis and management, there are no standard guidelines available for the diagnosis and management of PES, which has resulted in case-handling by non-endocrinologists and under-evaluation of hormones [6]. Primary empty sella syndrome (PES) is considered one of the causes of hypopituitarism. Structural causes of hypopituitarism like pituitary adenoma, pituitary abscess, apoplexy, and traumatic brain injury are associated with sequential pattern of hormonal deficiencies starts with Usually, the development of hypopituitarism occurs in a sequence of loss of function of the somatotrophic axis, subsequently followed by gonadotrophin, thyrotrophic, and finally corticotrophic axis, due to the anatomical location of the somatotropic, gonadotropic, thyrotropic, and corticotropic cells, respectively. Whether this sequence is respected in empty sella or not is uncertain. Usually, low IGF-1 levels are the first sign of hypopituitarism, but normal levels should not exclude the presence of dysfunctions in other axes, particularly in patients with empty sella, for which the patterns of hormonal dysfunctions, if any, remains to be characterized [7]. Hormonal deficiencies in PES can follow similar pattern if underlying causes of PES are related to the mentioned structural pathology before the time of the diagnosis of PES. Hormonal deficiency can be isolated single hormone or multiple hormonal deficiencies affecting all the hypothalamic-pituitary axeses. Symptomatic hyperprolactinemia in PES is rare in general but some patients might present with symptoms related to high prolactin. Hormonal alteration in PES is an area of controversy and it is not clear cut in most of the studies and accurate biochemical diagnosis is advocated. The most important part in hormonal assessment is to do basal hormonal assessment and dynamic testing only if it is indicated.

The findings may point towards poor case referrals to endocrinologists and sub-optimal assessment of hormonal profile. Evidence suggests that PES can mostly be an incidental finding without any symptoms [8]. In our experience, PES was mostly, also, an incidental finding diagnosed during radiological imaging of brain studies and hence not all patients had undergone endocrine evaluation. Gross under-evaluation of hormonal assessment is one of the significant findings of the present research, although almost 42% of the patients were symptomatic and presented with variable clinical features. This, however, precludes the possibility of overlooking PES as an incidental finding and demonstrates the role of endocrinologists being the cornerstone in the assessment and management of hormonal deficiencies.

In addition, the challenges of the diagnoses of central hormonal deficit since the central hormones may either be inappropriately normal, or mildy below the lower normal range may also lead to underdiagnosis of empty sella-related hormonal dysfunctions.

### Clinical presentation

In the current study, PES was almost four times more common among women establishing female preponderance, which is widely supported by other studies [9]. Although headache was the most common presenting symptom in our study, yet only 30% reported it in comparison to almost 70–90% of PES patients in other studies [10]. However, headache has not been though directly associated with PES, idiopathic intracranial hypertension (IIH) has long been the attributed cause, in addition to other co-existing symptoms like visual disturbances and orbital findings [11]. Saindaine and his colleagues evaluated incidental PES on MRI with IIH and documented headache as non-specific to PES but related IIH to be a significant determinant of headaches [12]. Visual disturbance was the second most common complaint presented in our study. Transient visual disturance, nystagmus, diplopia and blurred vision were some of the commonly reported visual impairment. Around 2.6% were diagnosed to have optic nerve abnormalities like compression, atrophy or neuritis. Most of the visual defects are a result of the involvement of optic nerve, which means that PES is more than just an incidental finding [13]. As identified by Saindaine and his team, visual defects cannot be characteristic of incidental PES, without eliminating the role of IIH. Although other studies have reported a lower prevalence of visual defects, the present finding suggests the importance of an ophthalmic assessment of PES patients with visual field defects [14].

Menstrual irregularities, galactorrhea and sexual disturbances were highly under-reported. But on the other hand, in contrast to our study, an Italy research reported a higher prevalence of menstrual and sexual dysfunction [15]. Non-assessment of menstrual irregularities is probably the main reason for the low prevalence in the present study. Other causes include the possibility of social stigma, fear, and discomfort feeling in reporting the symptoms. The highly conservative nature of Saudi society may also play an important role in under-reporting these symptoms. Concerning other comorbidities, diabetes and hypertension were the two most frequently reported conditions affecting 11% of the study patients.

### Overall hormonal assessment

The pituitary function in PES has been previously considered normal [4, 15], while many recent studies have been reporting growing evidence of impaired pituitary function [5, 16]. Pituitary insufficiency has enormous variations, ranging from isolated hormone deficiency to complete deficiency like panhypopituitarism. The present study found a gross under-evaluation of the hormonal profile. In the overall sample, only 10% were assessed for LH and FSH, 8% for cortisol, 5% for testosterone, 2% for estradiol and less than 1.3% were assessed for growth hormone. Thyroid and prolactin hormones assessment were slightly higher with 40 and 14%, respectively. Although the latter two being the most prevalent endocrine abnormalities, the case referral and hormonal assessment were still considered sub-optimal. The findings suggest under-evaluation leading to under-reporting the prevalence of pituitary hormones insufficiency that is of significant concern.

### Endocrine dysfunction and clinical management of cases

One of the principal finding of the study is the gross difference in the assessment of the hormones and the prevalence of deficiencies among those evalauated by the physicians and the endocrinologists. Differences in prevalence of hormonal deficiencies concerning the gonadal axis and somatotrophic axis were markedly observed followed by adrenal hormones and thyrotrophic axis. These findings reflect on the major consequences of poor refrerral and under-assessment of the cases by the general physicians. Most of the patients diagnosed with primary empty sella syndrome presented with one or multiple hormonal deficiency at the same time as the initial evaluation which made it difficult to follow on the sequences of the deficiency of all the hormones.

### Gonadal axis dysfunction

Further analysis of individual parameters showed marked endocrine dysfunction. A high prevalence of hypogonadism was noted in women, while adrenal insufficiency was common in men. The gonadal profile of PES patients in the present study showed marked variations in the assessment. Only 2.5% of the patients were referred for gonadal profile evaluation by the general physicians, while endocrinologists evaluated 52%. Among those assessed, almost 43% were affected by hypogonadismin the patients seen by endocrinologists in stark contrast to 6.6% in the other group. Similar findings were reported in the evaluation of adrenal hormones. The alarmingly high rates of low case referrals for hormone assessment increases the risk of under-diagnosis of associated abnormalities. Therefore, it becomes imperative for the subjective evaluation of these patients for complete hormonal assessment and diagnosis of hormone dysfunction in the PES patients.

### Growth hormone evaluation

Research studies have reported growth hormone deficiency to be the most common endocrine dysfunction in PES [17, 18]. The present study could, however, not correlate this finding with other studies since there is gross under-evaluation of IGF-1 hormone. Of the five women patients assessed for IGF-1, four patients had values less than 90 ng/ml, which is below the normal recommended standards for adults and all five patients had short stature, which means that IGF-1 is not being evaluated unless supported by clinical symptoms. While on the other hand, substantial evidence has suggested a high possibility of involvement of somatotroph cells of the anterior pituitary lobe due to its anatomic juxtaposition and predisposition for intrasellar pressure changes. The prevalence of GH hormone deficiency and its clinical implications among the PES patients was studied by Poggi and his team who found high levels of GH deficiency, thereby emphasizing the importance of screening for GH deficiency among the PES patients [19]. Hence optimal evaluation of growth hormone deficiency is essential to diagnose and correct the growth status in the case of children and maintain the essential levels of growth hormone for adults.

### Lactotrophic axis dysfunction

While hyperprolactinemia has long been associated with PES and considered one of the highly prevalent endocrine abnormality [20], our study also showed consistent results with high prolactin levels among men and women with a slight predisposition towards men though not significant. Since the prolactin levels were abnormally high in two patients with values greater than 5000 and 9000 ng/ml they have had a diagnosis of prolactinoma.

### Abnormalities of thyrotrophic axis

Concerning patients with thyroid dysfunction, 46 patients were known cases of hypothyroidism and 33 patients were newly diagnosed with low TSH and FT4 and put on hormone therapy. One male hypothyroid patient, aged 31 years had a concomitant history of Burkitts lymphoma and Behcet’s disease while diagnosed with PES as an incidental finding. The present study showed hypothyroidism to be more common in women than in men. The study found large proportion of the patients not being evaluated for thyroid profile. This may not reflect on the true prevalence of hypothyroidism and may, in fact, lead to underestimation of the disorder. Studies have linked PES with autoimmune thyroid disease and have well documented the importance of thyroid assessment as a routine [21]. The present study highlights the potential need for cautious endocrine assessment and case referrals to endocrinologists.

### Cluster of abnormalities

Of the total study patients, 19% had some degree of hormonal deficiencies related to hypopituitarism and six patients were diagnosed with panhypopituitarism. It is not uncommon to find other abnormalities in the MRI scans of the pituitary that is not related to PES diagnosis like incidental pituitary adenoma or pituitary aneurysm. However, it is noteworthy to mention that although beyond the scope of the present study, 76 cases of pituitary adenoma were identified on the MRI scans.

### Limitations

This study is from single-center and hence may lack generalizability, which may be considered as one of the limitations of the study. However, it may still serve the purpose of documenting literature from the region of Saudi Arabia. By linking similar registries from different regions across Saudi Arabia, a representative national sample may be obtained, enabling a better understanding of the clinical presentation and management of patients with primary empty sella in Saudi Arabia.

## Conclusion

The findings of the study denote PES to be more than an incidental finding since high prevalence of co-existing endocrine abnormalities were found. Most of the cases are not properly evaluated by non-endocrinologists due to lack of appropriate loop feedback from the primary gland to the pituitary which is part of normal physiological response to the absence of primary gland function and make it difficult and challenging to non-endocrinologist to recognize the above abnormalities in different axes based on the available normal range values of the hormones. Lack of referral to endocrinologists and gross under-evaluation of hormonal assessment pointed towards poor clinical management and risk of being not replaced for the deficit hormones. This is our main concern with considerable clinical implications.

### Clinical implications

Clinical implications of the present study include concerns regarding not referring the suspected cases to the experts in the field of endocrinology for further diagnosis and management. There is a substantial need for formulation of a standard protocol for diagnosis and follow-up by a multi-disciplinary team of experts involving ophthalmologists and neurologists in addition to the endocrinologists advocated for holistic management of PES, hormonal dysfunction and all abnormalities related to it.

## Data Availability

The complete set of data supporting our findings can be found in the hospital’s radiology department registry. The datasets generated and/or analyzed during the current study are not publicly available due to the confidentiality of information but are available from the corresponding author on reasonable request.

## Abbreviations

*ACTH*: Adrenocorticotropic Hormone
*ADH*: Anti Diuretic Hormone
*CSF*: Cerebrospinal Fluid
*CT*: Computed Tomography
*ES*: Empty Sella
*FSH*: Follicle Stimulating Hormone
*FT4*: Free Thyroxine
*GH*: Growth Hormone
*IGF-I*: Insulin-like Growth Factor
*IRB*: Institution’s Review Board
*LH*: Luteinizing Hormone
*MRI*: Magnetic Resonance Imaging
*PES*: Primary Empty Sella
*SD*: Standard Deviation
*TSH*: Thyroid Stimulating Hormone
